# Polymorphism of prion protein gene (PRNP) in Nigerian sheep

**DOI:** 10.1080/19336896.2023.2186767

**Published:** 2023-03-09

**Authors:** Adeniyi C. Adeola, Semiu F. Bello, Abdussamad M. Abdussamad, Akanbi I. Mark, Oscar J. Sanke, Anyebe B. Onoja, Lotanna M. Nneji, Nasiru Abdullahi, Sunday C. Olaogun, Lawal D. Rogo, Godwin F. Mangbon, Shamsudeen L. Pedro, Manasseh P. Hiinan, Muhammad M. Mukhtar, Jebi Ibrahim, Hayatu Saidu, Philip M. Dawuda, Rukayya K. Bala, Hadiza L. Abdullahi, Adebowale E. Salako, Samia Kdidi, Mohamed Habib Yahyaoui, Ting-Ting Yin

**Affiliations:** a State Key Laboratory of Genetic Resources and Evolution & Yunnan Laboratory of Molecular Biology of Domestic Animals, Kunming Institute of Zoology, Chinese Academy of Sciences, Kunming, China; b Sino-Africa Joint Research Center, Chinese Academy of Sciences, Kunming, China; c Centre for Biotechnology Research, Bayero University, Kano, Nigeria; d Department of Animal Genetics, Breeding and Reproduction, College of Animal Science, South China Agricultural University, Guangzhou, China; e Department of Veterinary Physiology and Biochemistry, Faculty of Veterinary Medicine, Bayero University, Kano, Nigeria; f Ministry of Agriculture and Rural Development, Secretariat, Ibadan, Nigeria; g Taraba State Ministry of Agriculture and Natural Resources, Jalingo, Nigeria; h Department of Virology, College of Medicine, University of Ibadan, Ibadan, Nigeria; i Department of Ecology and Evolutionary Biology, Princeton University, Princeton, NJ, USA; j Department of Biochemistry, Faculty of Basic Medical Sciences, College of Health Sciences, Bayero University, Kano, Nigeria; k Department of Veterinary Medicine, Faculty of Veterinary Medicine, University of Ibadan, Ibadan, Nigeria; l Department of Medical Laboratory Science, Faculty of Allied Health Sciences, College of Health Sciences, Bayero University, Kano, Nigeria; m Division of Veterinary Office, Serti, Nigeria; n Small Ruminant Section, Solomon Kesinton Agro-Allied Limited Iperu-Remo, Ogun State, Nigeria; o Department of Veterinary Surgery and Theriogenology, College of Veterinary Medicine, University of Agriculture Makurdi, Makurdi, Nigeria; p Department of Animal Science, Faculty of Agriculture, National University of Lesotho, Lesotho, Southern Africa; q Department of Animal Science, Faculty of Agriculture, University of Ibadan, Ibadan, Nigeria; r Livestock and Wildlife Laboratory, Institut des Régions Arides, Université de Gabes, Medenine, Tunisia

**Keywords:** Nigeria, polymorphism, prion protein gene, scrapie, sheep, susceptibility

## Abstract

Polymorphism of the prion protein gene (*PRNP*) gene determines an animal’s susceptibility to scrapie. Three polymorphisms at codons 136, 154, and 171 have been linked to classical scrapie susceptibility, although many variants of *PRNP* have been reported. However, no study has investigated scrapie susceptibility in Nigerian sheep from the drier agro-climate zones. In this study, we aimed to identify *PRNP* polymorphism in nucleotide sequences of 126 Nigerian sheep by comparing them with public available studies on scrapie-affected sheep. Further, we deployed Polyphen-2, PROVEAN, and AMYCO analyses to determine the structure changes produced by the non-synonymous SNPs. Nineteen (19) SNPs were found in Nigerian sheep with 14 being non-synonymous. Interestingly, one novel SNP (T718C) was identified. There was a significant difference (*P* < 0.05) in the allele frequencies of *PRNP* codon 154 between sheep in Italy and Nigeria. Based on the prediction by Polyphen-2, R154H was probably damaging while H171Q was benign. Contrarily, all SNPs were neutral via PROVEAN analysis while two haplotypes (HYKK and HDKK) had similar amyloid propensity of *PRNP* with resistance haplotype in Nigerian sheep. Our study provides valuable information that could be possibly adopted in programs targeted at breeding for scrapie resistance in sheep from tropical regions.

## Introduction

Prion proteins (*PRNP*) play a crucial role in Transmissible Spongiform Encephalopathies (TSEs), which are distinctive diseases that can be inherited and contagious [1–4]. In domestic animals, TSEs include scrapie in ovine and caprine, bovine spongiform encephalopathy (BSE) in cattle, and feline spongiform encephalopathy (FSE) in cats etc [5–12]. The epidemic form of prion protein (PrPSc) has diverse structural dynamics compared to cellular prion (PrPC), and specific points of prion protein were found related to the conversion of PrCP to PrPSc [13–16].

The susceptibility of animals to scrapie is predominantly controlled by polymorphism in the *PRNP* gene [17]. Based on this, different single nucleotide mutations have been identified at codons 136 (A > V), 154 (*R* > H), and 171 (*R* > Q/H) of *PRNP* gene [18,19]. Amino acids at codons 141 and 154 have been found to be associated with various forms of classical scrapie through modification of the configuration of prion protein [20,21]. Changes at codon 136 from A to V has been reported to increase susceptibility to scrapie while variations at codon 171 from Q to R cause resistance in sheep [18,22].

It was believed that the ancestral *PRNP* gene was ARQ/ARQ (ARQ/ARQ wild type) in sheep [23]. There are six primary forms of the wild-type allele which include ARQ, VRQ, AHQ, ARH, ARK, and ARR [24]. Also, the genotypes associated with the admixture of these alleles are classified into five groups based on their degrees of resistance to scrapie [25]. Recent studies have shown that amino acid substitution in some nucleotide positions were associated with resistance to scrapie and TSE in ARQ/ARQ sheep [26].

Several studies have reported the polymorphism of the *PRNP* gene in sheep of different breeds or economic traits across different countries such as in testis-specific *PRNP* gene related to phenotypes of Chinese and Mongolian sheep [27], phenotypic traits in different breeds of Chinese sheep [28], Xinjiang local breeds in China [29], and Tan sheep of Ningxia, China [30]. In addition, studies on *PRNP* gene have been carried considering different traits such as milk traits in Latxa dairy [31], reproductive traits in breeds of German sheep [32], Baltic breeds [33], pathogenesis and neuropathological phenotype [34], Rasa Aragonesa (Spanish breed) [7], milk production traits in Spanish Churra [35], Greece [36], Ireland [37], Brazil [38], Portugal [39], Italy [40], Turkey [41,42], and Palestine native sheep breed [42]. Moreover, polymorphism studies of the *PRNP* gene in sheep from Inner Mongolian [43] and Northwest China [44] have been reported. In Africa, only few polymorphism studies on *PRNP* gene in sheep have been reported in Algeria [45], Ethiopia [46], Morocco [47], and four Western African sheep population (i.e. Burkina-Sahel, Djallonke, Mossi and Touareg) [48].

Nigerian sheep are reared in the drier agro-climatic zones of the country with an estimated population of 27 million [49]. There are four major breeds of Nigerian sheep: Yankasa, Uda, Balami, and West Africa Dwarf [50]. Sheep have socio-economic values in Nigeria as they form part of the livelihoods of small ruminant farmers that are majorly rural dwellers [51]. Environmental factors of Nigeria impose health-related risks on the lives of human and animals which require urgent attention [52].

Several studies have been carried out on Nigerian sheep using genetic techniques such as microsatellite DNA polymorphism [53–56], polymorphism study on Myostatin (MSTN) [57], and transferrin and haemoglobin [58]. The survey conducted on the occurrence of scrapie in Jos within the central part of Nigeria [59] is not sufficient to conclude on the absence of scrapie in Nigerian sheep. There is no report on polymorphism of the *PRNP* gene in Nigerian sheep, despite its significant effects on scrapie, the economic importance of Nigerian sheep, and transmission of the diseases from animals to humans.

Herein, we examined the genotype and allele frequencies of *PRNP* polymorphisms in 126 Nigerian sheep and compared the sampled population with previous studies on scrapie-affected animals in different breeds of sheep. Subsequently, we evaluated the linkage disequilibrium (LD) and analysed haplotypes of the *PRNP* polymorphisms in Nigerian sheep. Finally, we computed the biological impact, which includes the protein structure and functions of nonsynonymous SNPs via PolyPhen-2, PROVEAN, and AMYCO investigations.

## Materials and methods

### Blood sample collection and DNA extraction

We collected 10 ml of blood samples from 126 sheep (61 females and 65 males) from four different states in Nigeria namely, Kaduna State (*n* = 18 female; *n* = 15 male), Katsina State (*n* = 7 female; *n* = 8 male), Sokoto State (*n* = 9 female; *n* = 15 male), and Taraba State (*n* = 26 female; *n* = 28 male) (Supplementary Fig S1). During sample collection, we excluded sheep with close relationships after obtaining information from the herders. The whole blood samples were stored at −20 ◦C prior to DNA extraction. Genomic DNA was extracted at Kunming Institute of Zoology, Chinese Academy of Sciences (CAS) using the phenol-chloroform method [60]. This was followed by the quantification using the Thermo Scientific™ NanoDrop 2000 spectrophotometer to evaluate the purity of the obtained DNA. Further, the quality of the total genomic DNA was checked by running gel electrophoresis using a 2% agarose gel with a 2 Kilobase (kb) DNA ladder marker. The 126 Nigerian sheep samples were then sequenced using the Sanger method.

### Polymerase chain reaction (PCR) and DNA sequencing

To detect the polymorphic sites of the *PRNP* gene in sheep. PCR was performed with one pair of primers: *PRNP*-Forward (5’- CATTTATGACCTAGAATGTTTATAGCTGATGCCA −3’) and *PRNP*-Reverse (5’- TTGAATGAATATTATGTGGCCTCCTTCCAGAC −3’) [45]. The 25 µl PCR mixture and sequencing reactions contained 1 μl of genomic DNA, 10 pmol of each primer, 2.5 mM dNTPs, and 5 units of Takara Taq DNA polymerase in a 10 pmol reaction buffer containing 1.5 mM MgCl2.

The PCR condition was as follows: 96°C for 5 min, 35 cycles of denaturation at 96°C for 30 s, 57°C for 15 s, 72°C for 1 min 30 s, and final extension of 72°C at 4 min. PCR products were purified for sequencing analysis with a QIAquick Gel Extraction Kit (Qiagen, Valencia California, USA). The PCR products were bidirectionally sequenced in an ABI 3730×L sequencer (Applied Biosystems, Foster City, California, USA).

### Statistical analysis

Hardy Weinberg Equilibrium (HWE), Linkage Disequilibrium (LD) and haplotype distributions of the *PRNP* gene in Nigeria sheep were performed using DNA SNP Version 6.12.03 [61]. The genotype differences, allele, and haplotype frequencies of the *PRNP* gene were analysed by chi-square test (χ2) or Fisher’s exact test using SPSS v21.0.

### Evaluation of nonsynonymous SNPs in the caprine prion protein

Fourteen (14) nonsynonymous SNPs of the *PRNP* gene were evaluated using PolyPhen-2 (http://genetics.bwh.harvard.edu/pph2/) and PROVEAN (http://provean.jcvi.org/index.php). Furthermore, we determined the Amyloid propensity of ovine prion protein using alleles of *PRNP* SNPs via AMYCO (http://bioinf.uab.cat/amyco) [62]. AMYCO is the algorithm for predicting amyloid fibril propensity from amino acid sequences.

## Results

### Identification of polymorphic sites of the PRNP gene in 126 Nigerian sheep

To achieve this, the open reading frame (ORF) of the *PRNP* gene in 126 Nigerian sheep was sequenced. The ORF contained 771 bp in length and occupied similar position with the *PRNP* gene of *Ovis aries* retrieved from the NCBI database (Gene ID:EF153678). In this study, we identified 19 SNPs, including 14 nonsynonymous SNPs being the T718C a novel SNP. (Supplementary Table S1 and Supplementary Figure S2). Table 1 shows the genotype and allele frequencies of the 13 non-synonymous SNPs of *PRNP* in Nigerian sheep. Interestingly, all genotype frequencies of the identified SNPs conform to Hardy-Weinberg Equilibrium (HWE).

**Table 1.** Genotype and allele frequencies of 19 PRNP polymorphism in Nigerian sheep.

	Genotype frequency, n (%)			Allele frequency, n (%)		HWE
G36T	GG	GT	TT	G	T	
12 V	0 (0.00)	0 (0.00)	126 (1.00)	0 (0.00)	252 (100.00)	
A302G	AA	AG	GG	A	G	
Q101R	125 (0.99)	0 (0.00)	1(0.01)	250 (99.21)	2 (0.79)	<0.0001
C335T	CC	CT	TT	C	T	
T112M	0 (0.00)	0 (0.00)	126 (1.00)	0 (0.00)	252 (100.00)	
G346C	GG	GC	CC	G	C	
A116P	123 (0.98)	0 (0.00)	3 (0.02)	246 (97.62)	6 (2.38)	<0.0001
A379G	AA	AG	GG	A	G	
S127G	0 (0.00)	0 (0.00)	126 (1.00)	0 (0.00)	252 (100.00)	<0.0001
A385G	AA	AG	GG	A	G	
S129G	0 (0.00)	0 (0.00)	126 (1.00)	0 (0.00)	252 (100.00)	
C414T	CC	CT	TT	C	T	
138S	125 (0.99)	0 (0.00)	1(0.01)	250 (99.21)	2 (0.79)	<0.0001
A428G	AA	AG	GG	A	G	
H143R	110 (0.87)	0 (0.00)	16 (0.13)	220 (87.30)	32 (12.70)	<0.0001
C451G	CC	CG	GG	C	G	
R151G	125 (0.99)	0 (0.00)	1(0.01)	250 (99.21)	2 (0.79)	<0.0001
G461A	GG	GA	AA	G	A	
R154H	121 (0.96)	0 (0.00)	5 (0.04)	242 (96.03)	10 (3.97)	<0.0001
A512G	AA	AG	GG	A	G	
H171Q	124 (0.98)	0 (0.00)	2 (0.02)	248 (98.41)	4 (1.59)	<0.0001
T513G	TT	TG	GG	T	G	
H171Q	0 (0.00)	0 (0.00)	126 (1.00)	0 (0.00)	252 (100.00)	
T514G	TT	TG	GG	T	G	
Y172D	118 (0.94)	0 (0.00)	8 (0.06)	236 (93.65)	16 (6.35)	<0.0001
G526A	GG	GA	AA	G	A	
N176K	0 (0.00)	0 (0.00)	126 (1.00)	0 (0.00)	252 (100.00)	
C528A	CC	CA	AA	C	A	
N176K	102 (0.81)	0 (0.00)	24 (0.19)	204 (80.95)	48 (19.05)	<0.0001
C691A	CC	CA	AA	C	A	
230Q	39 (0.31)	0 (0.00)	87 (0.69)	78 (30.95)	174 (69.05)	<0.0001
G711C	GG	GC	CC	G	C	
237 L	69 (0.55)	0 (0.00)	57 (0.45)	138 (54.76)	114 (45.24)	<0.0001
T718C	TT	TC	CC	T	C	
S240P	124 (0.98)	0 (0.00)	2 (0.02)	244 (98.39)	4 (1.61)	<0.0001
T750C	TT	TC	CC	T	C	
250 L	0 (0.00)	0 (0.00)	126 (1.00)	0 (0.00)	252 (100.00)	

HWE: Hardy-Weinberg equilibrium.

Also, we examined the LD among the 19 SNPs detected with others from previous studies using Lewontin’s D’ (|D’|) values (Table 2). The majority of the SNPs showed negative LD ranging from −0.018 to −0.188, except c.711 G > C which showed strong positive LD values of 0.135 and 0.095 with some SNPs.

**Table 2.** Linkage Disequilibrium (LD) of nineteen (19) *PRNP* polymorphisms in Nigerian sheep.

	G36T 12 V	A302G Q101R	C335T T112M	G346C A116P	A379G S127G	A385G S129G	C414T 138S	A428G H143R	C451G R151G	G461A R154H	A512G H171Q	T513G H171Q	T514G Y172D	G526A N176K	C528A N176K	C691A 230Q	G711C 237 L	T718C S240P	T750C 250 L
G36T 12 V	-	−0.008	−0.008	−0.014	0.704	−0.008	−0.011	−0.0333	−0.008	−0.018	−0.011	0.704	−0.023	1.000	−0.042	0.132	−0.082	−0.011	−0.008
A302G Q101R	-	-	−0.008	−0.014	−0.011	−0.008	0.704	−0.033	−0.008	−0.018	−0.011	−0.011	−0.023	−0.008	−0.042	−0.059	0.095	0.704	−0.008
C335T T112M	-	-	-	−0.014	−0.011	1.000	−0.011	−0.033	−0.088	−0.018	−0.011	−0.011	−0.023	−0.008	−0.042	−0.059	0.095	−0.011	−0.008
G346C A116P	-	-	-	-	−0.019	−0.014	−0.019	−0.058	−0.014	−0.031	−0.019	−0.019	−0.039	−0.014	−0.073	0.120	−0.142	−0.019	−0.014
A379G S127G	-	-	-	-	-	−0.011	−0.016	−0.047	−0.011	−0.025	−0.016	0.492	−0.032	0.704	−0.059	0.052	0.010	−0.016	−0.011
A385G S129G	-	-	-	-	-	-	−0.011	−0.033	−0.008	−0.018	−0.011	−0.011	−0.023	−0.008	−0.042	−0.059	0.095	−0.011	−0.008
C414T 138S	-	-	-	-	-	-	-	−0.047	−0.011	−0.025	−0.016	−0.016	−0.032	−0.011	−0.059	−0.083	0.135	0.492	0.704
A428G H143R	-	-	-	-	-	-	-	-	−0.033	−0.075	−0.047	−0.047	0.001	−0.033	−0.058	−0.250	0.264	−0.047	−0.033
C451G R151G	-	-	-	-	-	-	-	-	-	0.440	−0.011	−0.011	−0.023	−0.088	−0.042	0.132	−0.082	−0.011	−0.008
G461A R154H	-	-	-	-	-	-	-	-	-	-	−0.025	−0.025	−0.051	−0.018	−0.095	0.040	−0.185	−0.025	−0.018
A512G H171Q	-	-	-	-	-	-	-	-	-	-	-	−0.016	−0.032	−0.011	−0.059	−0.083	0.135	−0.016	−0.011
T513G H171Q	-	-	-	-	-	-	-	-	-	-	-	-	−0.032	0.704	0.102	0.052	−0.116	−0.116	−0.011
T514G Y172D	-	-	-	-	-	-	-	-	-	-	-	-	-	−0.023	0.043	−0.171	0.084	−0.032	−0.023
G526A N176K	-	-	-	-	-	-	-	-	-	-	-	-	-	-	−0.042	0.132	−0.082	−0.011	−0.008
C528A N176K	-	-	-	-	-	-	-	-	-	-	-	-	-	-	-	−0.188	0.196	−0.059	−0.042
C691A 230Q	-	-	-	-	-	-	-	-	-	-	-	-	-	-	-	-	−0.584	−0.083	−0.059
G711C 237 L	-	-	-	-	-	-	-	-	-	-	-	-	-	-	-	-	-	0.135	0.095
T718C S240P	-	-	-	-	-	-	-	-	-	-	-	-	-	-	-	-	-	-	−0.011
T750C 250 L	-	-	-	-	-	-	-	-	-	-	-	-	-	-	-	-	-	-	-

Haplotype frequency of the 14 *PRNP* nonsynonymous SNPs were further examined as shown in Table 3. Based on the haplotype analysis, we found eight (8) major haplotypes with the haplotype QMAGGHRRQQYNNS having the highest frequency (58.0%) followed by QMAGGRRRQQYNNS (13.7%) and QMAGGHRRQQDKKS (1.5%).

**Table 3.** Haplotype frequencies of fourteen (14) non-synonymous single nucleotide polymorphism of *PRNP* gene in Nigerian sheep.

Haplotypes	A302G	C335T	G346C	A379G	A385G	A428G	C451G	G461A	A512G	T513G	T514G	G526A	C528A	T718C	N = 262
	Q101R	T112M	A116P	S127G	S129G	H143R	R151G	R154H	H171Q	H171Q	Y172D	N176K	N176K	S240P	
Haplotype 1	Q	M	A	G	G	H	R	R	Q	Q	Y	N	N	S	2 (0.0076)
Haplotype 2	Q	M	A	G	G	H	R	R	Q	Q	Y	N	N	S	62 (0.2366)
Haplotype 3	Q	M	A	G	G	H	R	R	Q	Q	Y	N	N	S	44 (0.1679)
Haplotype 4	Q	M	A	G	G	H	R	R	Q	Q	Y	K	K	S	8 (0.0305)
Haplotype 5	Q	M	A	G	G	H	R	R	Q	Q	Y	K	K	S	24 (0.0916)
Haplotype 6	Q	M	A	G	G	R	R	R	Q	Q	Y	N	N	S	6 (0.0229)
Haplotype 7	Q	M	A	G	G	R	R	R	Q	Q	Y	N	N	S	20 (0.0763)
Haplotype 8	Q	M	A	G	G	H	R	R	Q	Q	D	K	K	S	4 (0.0153)
Others															92 (0.3511)

## Estimation of potential scrapie vulnerability in Nigerian sheep

To estimate potential scrapie vulnerability in Nigerian sheep, we compared the genetic distribution of scrapie-related SNPs (R154H and S240P) between Nigerian sheep and scrapie-affected sheep in other countries. Previous studies related to SNPs of ovine *PRNP* gene were selected to estimate the susceptibility in Nigerian sheep [30,40].

**Figure 1.**
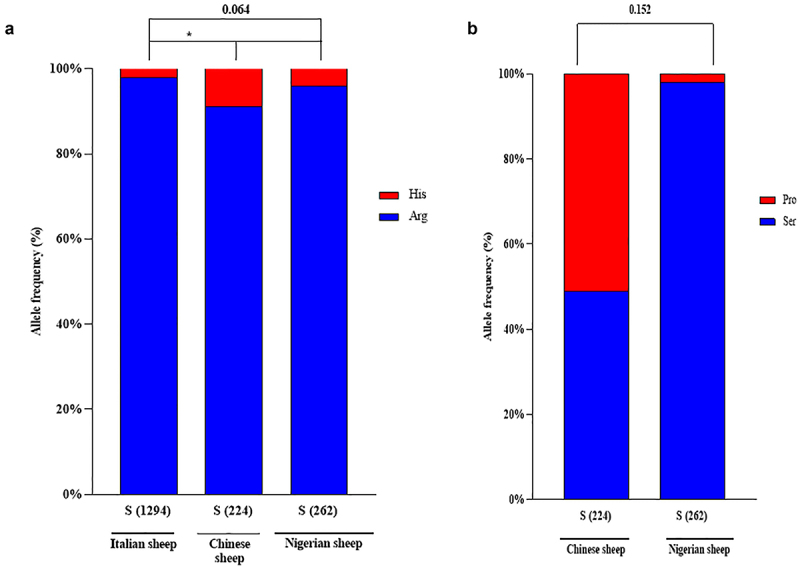
Comparisons of the allele frequencies of *PRNP* codons 154 and 240 in Central and Southern Italy, Chinese and Nigerian sheep. (a) Comparisons of the allele frequency of the *PRNP* codon 154 between Central and Southern Italy, Chinese sheep and Nigerian sheep [30,63] (b) Comparisons of the allele frequency of *PRNP* codon 240 between Chinese and Nigerian sheep [30].

There was a significant difference (*P<0.05*) in allele frequencies of scrapie-affected Italian sheep and healthy Nigerian sheep counterpart at *PRNP* codon 143 (Figure 1a). However, no significant (*P* = 0.0064) difference was recorded between the allele frequencies of scrapie-affected Chinese sheep and Nigerian sheep at codon 143 (Figure 1a).

Moreover, the allele frequencies of scrapie-affected Chinese and Nigerian sheep had no significant (*P* = 0.152) difference at codon 154 (Figure 1b).

## Comparison of haplotype and genotype frequencies at *PRNP* codons 143, 154 and 171 between scrapie-affected and healthy sheep

The ovine *PRNP* haplotypes of codons 143,154 and 171 in Nigerian sheep were used for comparison with other previously published works on *PRNP* in sheep. Moreover, the *PRNP* haplotypes were compared with those previously reported in two countries i.e. Italian and Spanish sheep [7,40]. In the considered countries, the ARR and AHQ haplotypes were significantly different between the scrapie-affected and healthy sheep (Table 4). In Italian and Spanish sheep, ARQ haplotype was present but was not in Nigerian sheep. The genotypes ARR/ARR and ARQ/ARQ were significantly different between the scrapie-affected and healthy sheep.

**Table 4.** Distributions of haplotype and genotype frequencies at *PRNP* codons 143, 154 and 171 between scrapie affected and healthy sheep.

	Italian sheep	Spanish sheep			Nigerian sheep
References	41	7			In this study
Haplotype	Healthy	Healthy	Scrapie-affected	*p-value*	Healthy
ARR	156.5 (12.09)	182.8 (10.10)	76.1 (7.05)	<0.00001	220 (87.30)
AHQ	10.1 (0.78)	25.2 (1.39)	8.4 (0.78)	<0.05	32 (12.70)
ARH	20.7 (1.60)	32.8 (1.81)	16.8 (1.56)		0 (0.00)
ARQ	198.9 (15.37)	510.8 (28.22)	391.5 (36.25)		0 (0.00)
VRQ	13.8 (1.07)	48.3 (2.67)	10.9 (1.01)		0 (0.00)
	Genotype				
ARR/ARR	64 (9.89)	44 (4.86)	17 (3.15)	<0.00001	110 (87.30)
ARR/AHQ	8.7 (1.34)	13 (1.44)	4 (0.74)		0 (0.00)
AHQ/AHQ	12.9 (1.99)	0 (0.00)	0 (0.00)		0 (0.00)
ARR/ARH	151.9 (23.48)	24 (2.65)	6 (1.11)		0 (0.00)
AHQ/ARH	0.7 (0.11)	0 (0.00)	1 (0.19)		0 (0.00)
ARR/ARQ	10.1 (1.56)	215 (23.76)	112 (20.74)		0 (0.00)
AHQ/ARQ	0 (0.00)	28 (3.09)	11 (2.04)		0 (0.00)
ARH/ARH	0.6 (0.09)	3 (0.33)	2 (0.37)		0 (0.00)
ARH/ARQ	25.1 (3.88)	30 (3.31)	14 (2.59)		0 (0.00)
ARQ/ARQ	98.3 (15.19)	350 (38.67)	310 (57.41)	<0.00001	16 (12.70)
ARR/VRQ	14 (2.16)	20 (2.21)	3 (0.56)		0 (0.00)
AHQ/VRQ	0 (0.00)	3 (0.33)	0 (0.00)		0 (0.00)
ARH/VRQ	2.2 (0.34)	5 (0.55)	1 (0.19)		0 (0.00)
ARQ/VRQ	0 (0.00)	56 (6.19)	17 (3.15)		0 (0.00)
VRQ/VRQ	0 (0.00)	7 (0.77)	1 (0.19)		0 (0.00)

## Assessment of nonsynonymous SNPs of the *PRNP* gene in Nigerian sheep

**Figure 2.**
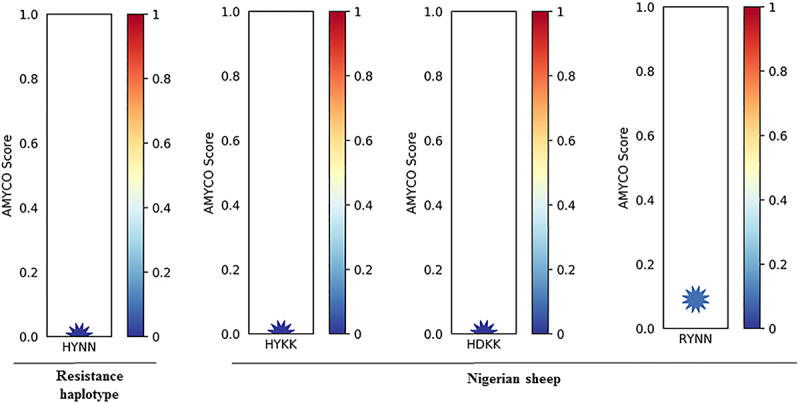
Prediction of amyloid propensity of sheep prion protein according to nonsynonymous SNPs. AMYCO predicted amyloid propensity as values from 0.0 to 1.0. The AMYCO scores<0.45 and>0.78 indicated low and high aggregation propensities of the protein, respectively. ‘HYNN’ indicates haplotype of serine allele at the codon 139, serine allele at the codon 146, histidine allele at the codon 154, and Isoleucine allele at the codon 193. ‘HYKK’ indicates haplotype of Arginine allele at the codon 139, asparagine allele at the codon 146, arginine allele at the codon 154, and threonine allele at the codon 193. ‘HDKK’ indicates haplotype of arginine allele at the codon 139, asparagine allele at the codon 146, histidine allele at the codon 154, and threonine allele at the codon 193. ‘RYNN’ indicates haplotype of arginine allele at the codon 139, asparagine allele at the codon 146, arginine allele at the codon 154, and isoleucine allele at the codon 193.

PolyPhen-2 is an online tool used to predict the outcome of an amino acid substitution caused by nonsynonymous SNPs on the structure and function of proteins [64]. Based on our polymorphism results, the effects of 14 non-synonymous SNPs identified were assessed via PolyPhen-2. Three different predictions were observed for the 14 non-synonymous SNPs which include benign: T112M (0.000), S127G (0.000), S129G (0.000), H143R (0.241), H171Q (0.000), N176K (0.002), probably damaging: A116P (1.000), R151G (1.000), R154H (0.998), Y172D (1.000), and possibly damaging: Q101R (0.621), S240P (0.827) as shown in Table 5. We predicted the biological impact of the nonsynonymous SNPs identified with PROVEAN [65]. Interestingly, the nonsynonymous SNPs of the *PRNP* gene identified in this study were predicted as ‘neutral’ (Table 5). Finally, we explored the amyloid propensity of ovine prion protein using the alleles of nonsynonymous SNPs. Based on this, we analysed the prion protein of these alleles and the HYNN haplotype was estimated with 0.00 values by AMYCO. Additionally, we evaluated the prion protein of Nigerian sheep. We classified amino acid sequences of *PRNP* in Nigerian sheep into three haplotypes (HYKK, HDKK and RYNN) considering the alleles of the 14 nonsynonymous SNPs. Based on AMYCO score, Haplotypes HYKK and HDKK when evaluated had a value of 0.00, while RYNN haplotype gave a value of 0.08 (Figure 2).

**Table 5.** Measurement of the effect of amino-acid substitutions of *PRNP* nonsynonymous SNPs in Nigerian sheep.

Position	AA_1_	AA_2_	Methods	Score	Prediction
101	Q	R	PolyPhen-2	0.621	Possibly damaging
			PROVEAN	−0.889	Neutral
112	T	M	PolyPhen-2	0.000	Benign
			PROVEAN	0.891	Neutral
116	A	P	PolyPhen-2	1.000	Probably damaging
			PROVEAN	−1.503	Neutral
127	S	G	PolyPhen-2	0.000	Benign
			PROVEAN	1.344	Neutral
129	S	G	PolyPhen-2	0.000	Benign
			PROVEAN	1.637	Neutral
143	H	R	PolyPhen-2	0.241	Benign
			PROVEAN	−1.532	Neutral
151	R	G	PolyPhen-2	1.000	Probably damaging
			PROVEAN	−2.354	Neutral
154	R	H	PolyPhen-2	0.998	Probably damaging
			PROVEAN	−0.491	Neutral
171	H	Q	PolyPhen-2	0.000	Benign
			PROVEAN	1.081	Neutral
172	Y	D	PolyPhen-2	1.000	Probably damaging
			PROVEAN	−1.277	Neutral
176	N	K	PolyPhen-2	0.002	Benign
			PROVEAN	−1.131	Neutral
240	S	P	PolyPhen-2	0.827	Possibly damaging
			PROVEAN	−0.89	Neutral

## Discussion

Scrapie is a fatal disease in sheep but the distribution of the PrP genotypes could serve as a means to prevent subsequent reoccurrence in breeding programme. This study analysed the nucleotide sequence of the *PRNP* gene in 126 Nigerian sheep and revealed 19 SNPs of which 14 were non-synonymous. Interestingly, one novel SNP (T718C) was identified.

The polymorphism of the *PRNP* gene at codons 136 [Alanine (A) or Valine (V)], 154 [arginine (A) or histidine (H)], and 171 [R, H or glutamine (G)] are highly related to vulnerability or resistance to scrapie in sheep [66–68]. The allelic variants (VRQ and ARQ) of *PRNP* at codons 136, 154 and 171 have been reported to correlate with high resistance to scrapie [69,70]. Moreover, the ARR allele was associated with low resistance to scrapie [71]. In contrast, the VRQ allele at the same codon had low survivability after exposure to scrapie [72]. Based on this, there is a need to develop breeding programs that will increase the frequency of the ARR allele [73,74]. It is believed that increasing such scrapie-resistant genotypes will enhance scrapie control.

In this present study, the non-synonymous substitutions i.e. Q101R, T112M, A116P, S127G, S129G, H143R, R151G, R154H, H171Q, Y172D, N176K, and S240P were identified (Table 5). The mutation at codon 127 was highly polymorphic similar to the trend detected in scrapie susceptibility in three native Ethiopian sheep breeds [46]. It has been reported that amino acids at codons 126 and 127 are found in highly conserved glycine-rich motif.

GAVVG126G127LGGYMLG could reduce development of prion disease via blockage of amyloid fibril formation [13]. In a previous, polymorphism at codon 127 was found to play a crucial role in the normal cellular function of *PRNP* [75]. Moreover, the genotypes at codon 171Q/K are also related to scrapie susceptibility [76].

The present study revealed that Nigerian sheep have different polymorphic sites mainly discovered at codons 112, 127, 129, 154, 171 and 176. Variations of *PRNP* at codons 112 and 129 have not been reported in sheep and their functions of codons 112 and 129 have not been established. However, we only detected polymorphism at codons 154 and 171, which are susceptible to classical scrapie in sheep.

Furthermore, PolyPhen-2 and PROVEAN were used to predict the impacts of 14 non-synonymous SNPs identified in this study. Using PolyPhen-2, three different predictions were observed for the 14 non-synonymous SNPs which include benign: T112M (0.000), S127G (0.000), S129G (0.000), H143R (0.241), H171Q (0.000), N176K (0.002), probably damaging: A116P (1.000), R151G (1.000), R154H (0.998), Y172D (1.000), and possibly damaging: Q101R (0.621), S240P (0.827). Although, all the 14 non-synonymous SNPs identified for the *PRNP* gene in Nigerian sheep populations were ‘(neutral or non-deleterious)’ using PROVEAN. The discrepancies in the prediction outcomes of the two software might be due to variations in algorithms that reproduce the effect on the position of the protein [64,77] and difference in their mode of analyses [78]. We investigated the amyloid propensity of sheep prion protein based on the alleles of nonsynonymous SNPs of the *PRNP* gene in sheep. We found out that the amyloid formation of *PRNP* in two of the haplotypes (HYKK and HDKK) was similar to the resistance haplotype (HYNN) to prion diseases in this study. However, one of the haplotypes (RYNN) had a higher degree of amyloid formation value than the resistance haplotype (Figure 2).

## Conclusion

In conclusion, the polymorphism identified in this study show that sheep in Nigeria are susceptible to scrapie because of the variations detected at codons 154 and 171. The information obtained about the variation in allelic frequencies of *PRNP* gene in Nigerian sheep could assist into designing valuable scrapie-resistance breeding projects especially for sheep in a tropical region like Nigeria, thereby reducing disease outbreaks caused by scrapie and the subsequent increase in cost of production.

## Supplementary Material

Supplemental Material

## Data Availability

Accession no. MZ463500 – MZ463625 https://www.ncbi.nlm.nih.gov/
