# Supplementary material for: Polymorphism of prion protein gene (PRNP) in Nigerian sheep
Source: Prion. 2023 Mar 9;17(1):44–54. doi: 10.1080/19336896.2023.2186767 (PMC10012947; doi:10.1080/19336896.2023.2186767)
Supplement: Supplemental Material [file KPRN_A_2186767_SM5394.zip › Supplementary Materials.docx]

**Supplementary Materials:**

Figure S1: Map of Nigeria showing the five sampled areas in this study

Figure S2: Electropherogram showing one of the individuals with novel SNP (T718C) when aligned with reference sequence (U67922).

Table S1: SNP sites for 126 Nigerian Sheep
