# Supplementary figures and images for: Polymorphism of prion protein gene (PRNP) in Nigerian sheep
Source: Prion. 2023 Mar 9;17(1):44–54. doi: 10.1080/19336896.2023.2186767 (PMC10012947; doi:10.1080/19336896.2023.2186767)

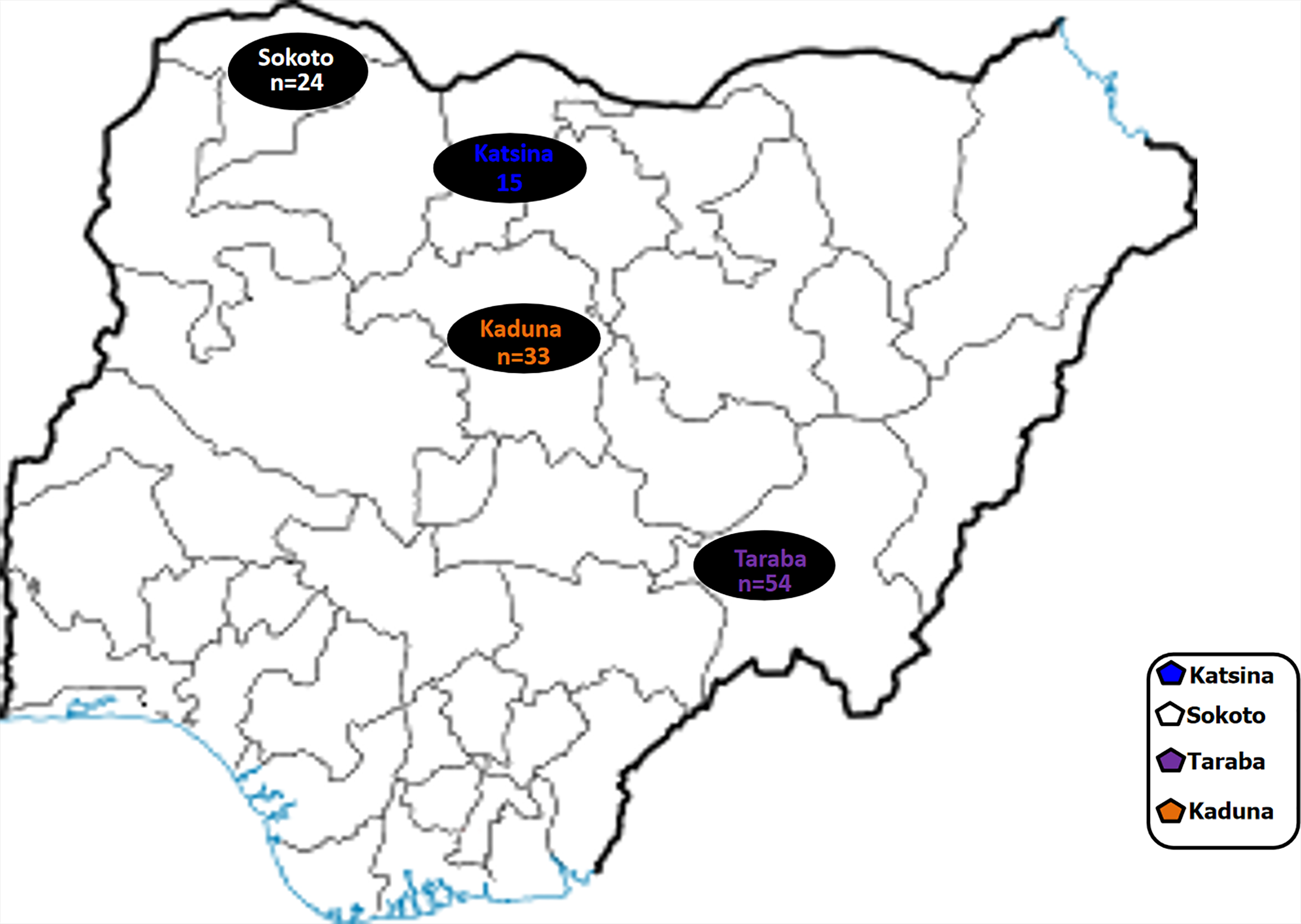

Supplement: Supplemental Material [file KPRN_A_2186767_SM5394.zip › Supplementary Fig 1_R1.tif]

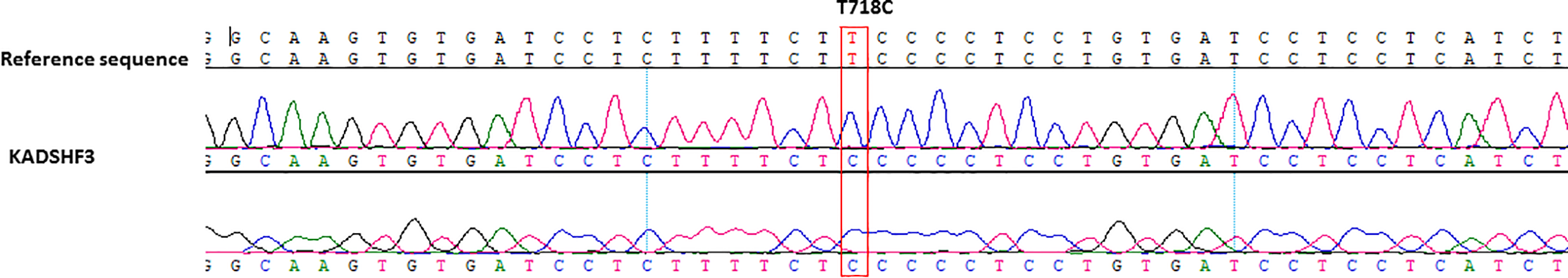

Supplement: Supplemental Material [file KPRN_A_2186767_SM5394.zip › Supplementary Fig 2_R1.tif]
